# Durvalumab consolidation therapy in patients with stage III small cell lung cancer after concurrent chemoradiation: a China-based cost-effectiveness analysis

**DOI:** 10.3389/fonc.2025.1643022

**Published:** 2025-11-05

**Authors:** Hui Zhang, Yuhang Liu, Bikun Cai, Mengyi Wang, Haonan Li, Hong Wang

**Affiliations:** ^1^ School of Medical Business, Guangdong Pharmaceutical University, Guangzhou, Guangdong, China; ^2^ Guangdong Health Economics and Promotion Research Centre, Guangdong Pharmaceutical University, Guangzhou, Guangdong, China; ^3^ Guangdong Health Economics and Health Promotion Research Centre, Guangdong Pharmaceutical University, Guangzhou, Guangdong, China; ^4^ School of Pharmaceutical Sciences, Peking University, Beijing, China; ^5^ International Research Centre for Medicinal Administration, Peking University, Beijing, China

**Keywords:** durvalumab, limited-stage small-cell lung cancer, Markov model, placebo, cost-effectiveness

## Abstract

**Background:**

Limited-stage small-cell lung cancer (LS-SCLC) has suboptimal long-term survival despite standard chemoradiotherapy. Durvalumab, an anti-PD-L1 antibody, demonstrated survival benefits in the ADRIATIC Phase III trial, but its cost-effectiveness in China remains uncertain. This study evaluates the economic value of durvalumab as consolidation therapy for LS-SCLC post-chemoradiotherapy.

**Methods:**

A Markov model was constructed using data from the ADRIATIC trial (NCT03703297), simulating three health states: progression-free survival (PFS), progressive disease (PD), and death state, transition probabilities were derived from trial outcomes. A 10-year horizon, 5.0% discount rate, and willingness-to-pay (WTP) thresholds (1-3×per capita gross domestic product (GDP): $12,569.82-$37,709.46/QALY) were applied. All costs were converted to unified currency using the average exchange rate of 1 USD = 7.11 CNY, based on exchange rates from 1 January 2024, to 31 October 2024.

**Results:**

The study results demonstrated that while durvalumab provided clinical benefits by extending quality-adjusted life years (QALYs) by 0.44 compared to placebo (2.24 vs. 1.80), its high cost resulted in poor cost-effectiveness within China’s healthcare system. The incremental cost reached $108,609.45, yielding an ICER of $245,591.59 per QALY, exceeding China’s standard willingness-to-pay thresholds. Sensitivity analyses revealed drug pricing as the most influential factor, where a 30% price reduction could improve the ICER by 30.3%. The negative incremental net monetary benefit (-$107,394.34) further confirmed the economic challenges. These findings suggest that despite its clinical advantages, durvalumab’s current pricing makes it economically unviable for routine use in China’s LS-SCLC treatment without substantial cost reductions or alternative reimbursement strategies.

**Conclusion:**

Durvalumab improves survival in LS-SCLC but lacks cost-effectiveness under current pricing in China. Drug costs and health utilities are critical determinants. Policy measures, such as price negotiation, risk-sharing agreements, or subgroup targeting, may enhance affordability. Balancing clinical benefits with economic burden is essential for optimizing durvalumab’s role in LS-SCLC management.

## 1 Introduction

Small cell lung Cancer (SCLC) is a highly aggressive neuroendocrine tumor, accounting for approximately 14.0% of all lung cancer cases, and is closely associated with smoking (with about 95.0% of patients having a history of smoking) (1, 2). SCLC is characterized by early metastasis, rapid proliferation, and susceptibility to drug resistance, with a 5-year survival rate of only 6.4% (3). Based on the extent of disease, SCLC is divided into limited-stage (LS-SCLC) and extensive-stage (ES-SCLC), with the latter accounting for 60%-70% of diagnosed patients (1). In traditional treatment, patients with limited-stage disease primarily receive concurrent chemoradiotherapy, while those with extensive-stage disease are treated with platinum-based chemotherapy combined with etoposide. However, the median overall survival (OS) is only about 10 months, and most patients relapse within 6 months after treatment (4).

In recent years, the introduction of immune checkpoint inhibitors has significantly transformed the treatment landscape for ES-SCLC. Based on the results of the CASPIAN and IMpower133 trials, PD-L1 inhibitors such as durvalumab and atezolizumab, in combination with chemotherapy, have been established as a new standard of first-line treatment (5–7). The CASPIAN trial demonstrated that the median OS in the durvalumab plus chemotherapy group was 13.0 months (compared to 10.3 months in the chemotherapy-alone group, HR = 0.73), with a 3-year OS rate increased to 17.6% (compared to 5.8% in the chemotherapy-alone group) (6, 7). Despite these improvements, the absolute survival benefit of immunotherapy remains relatively limited (an extension of 2–3 months), and some patients require treatment adjustments due to immune-related adverse reactions (such as pneumonia and autoimmune diseases) (5, 7).

Durvalumab is a selective, high-affinity humanized IgG1 monoclonal antibody that exerts anti-tumor effects by blocking the interaction between PD-L1 and PD-1 as well as CD80. In recent years, durvalumab has demonstrated significant efficacy and controllable safety in the treatment of various types of lung cancer. In the CASPIAN trial, durvalumab in combination with platinum–etoposide chemotherapy as first-line treatment for ES-SCLC significantly prolonged OS and progression-free survival (PFS) (8). The POSEIDON study further confirmed the efficacy of durvalumab combined with chemotherapy in metastatic non-small cell lung cancer (mNSCLC). Compared with chemotherapy alone, it significantly improved PFS and showed a trend toward prolonged OS (9). Additionally, the PACIFIC study evaluated the effect of durvalumab as consolidation therapy in patients with stage III unresectable NSCLC. The results showed that it significantly prolonged PFS and OS (10). The AEGEAN trial explored the application of durvalumab in the perioperative period for patients with resectable NSCLC. The results indicated that durvalumab combined with neoadjuvant chemotherapy significantly prolonged event-free survival (EFS) and increased the pathological complete response rate (pCR) (11). Most recently, the ADRIATIC trial investigated the efficacy of durvalumab as adjuvant therapy in LS-SCLC. The results showed that the durvalumab treatment group significantly prolonged OS and PFS compared with the placebo group (12).

These study results indicate that durvalumab has potential application value in the treatment of lung cancer at different stages and types, especially in improving patient survival prognosis. In recent years, China has been continuously advancing policy reforms to improve the accessibility of innovative drugs. The National Medical Products Administration (NMPA) has established breakthrough treatment drug and priority review procedures to accelerate the market entry of drugs with outstanding clinical value. The National Healthcare Security Administration (NHSA) has included several innovative immunotherapy drugs, including durvalumab, in the national medical insurance list through medical insurance negotiation mechanisms (13). However, as a high-value innovative drug with a relatively long treatment cycle, durvalumab has sparked widespread attention regarding its economic affordability and cost-effectiveness.

Pharmacoeconomic studies can systematically model the cost-effectiveness relationship of drugs in real clinical applications, helping clinicians, medical insurance providers, and policymakers make scientific decisions with limited health resources. Especially in the group of patients with LS-SCLC, who need intensive treatment and face a high risk of recurrence, clarifying the cost-effectiveness characteristics of durvalumab as consolidation therapy is of great significance for maximizing its clinical value and optimizing resource allocation.

To ensure scientific rigor, the analysis utilizes data derived from a registered clinical study (NCT03703297) that was first posted on ClinicalTrials.gov on September 27, 2018.

## 2 Materials and methods

### 2.1 Target population

The target population of this study consists of patients with limited-stage small-cell lung cancer (LS-SCLC) who have completed radical chemo-radiotherapy without disease progression. These patients still face a high risk of recurrence after standard treatment and require further consolidation therapy to prolong PFS and overall survival (OS). The characteristics of the target population include being in a mild disease state with no progression after radical chemoradiotherapy, and the need for a consolidation treatment that can significantly extend PFS and OS, while also considering the safety and economic affordability of the treatment.

The enrolled patients were derived from a multicenter, randomized, double-blind, phase 3 clinical trial comparing durvalumab (1,500 mg) with or without tremelimumab (75 mg for four doses only) to placebo, administered every 4 weeks for up to 24 months in limited-stage small-cell lung cancer patients who had not experienced disease progression after standard concurrent platinum-based chemoradiotherapy. The study enrolled 730 patients who were randomly assigned in a 1:1:1 ratio, with 264 patients allocated to the durvalumab group, 200 to the durvalumab-tremelimumab group, and 266 to the placebo group. Randomization was stratified according to disease stage (I or II vs. III) and receipt of prophylactic cranial irradiation (yes vs. no). All participants had completed concurrent chemoradiotherapy prior to enrollment. The trial was conducted across multiple centers, representing diverse populations.

All study procedures were rigorously performed in compliance with applicable ethical guidelines and regulatory requirements. The current analysis utilized data from the ADRIATIC phase III clinical trial (Cheng et al.) (13). Prior to study enrollment, written informed consent was obtained from all participating individuals or their legally authorized representatives. The phase 3 trial was conducted across multiple centers in Asia, Europe, and North and South America, representing diverse urban medical centers under various healthcare systems. For the purpose of this analysis, we established standardized baseline patient characteristics, including a median age of 62 years, with most patients being former or current smokers. Adverse events (AEs) were graded according to the Common Terminology Criteria for Adverse Events, version 4.03, and included in the analysis if they reached grade 3 or higher (G3+) in either treatment group (durvalumab or placebo). This study was carried out based on the phase 3 trial and did not involve other human participants, hence, there is no need for the approval of the independent ethics committee.

### 2.2 Model construction

Based on the ADRIATIC Phase III trial (NCT03703297), this study developed a Markov model to evaluate the cost-effectiveness of adjuvant durvalumab versus placebo in patients with LS-SCLC without progression after chemoradiotherapy (14), from the Chinese healthcare system perspective. The model employed a monthly cycle length and a 10-year time horizon, simulating 95% of clinical events. Health outcomes included life years (LYs), QALYs, and ICERs, with cost-effectiveness thresholds defined as 1–3 times China’s 2024 per capita GDP 12,569.82 CNY (89,371.42 USD)-37,709.46 CNY (268114.26 USD). Microsoft Excel 2019 was used for model implementation, and all costs were adjusted to 2024 US dollars (exchange rate: 1 USD = 7.11 CNY) using healthcare-specific inflation indices (10).

**Figure 1:**
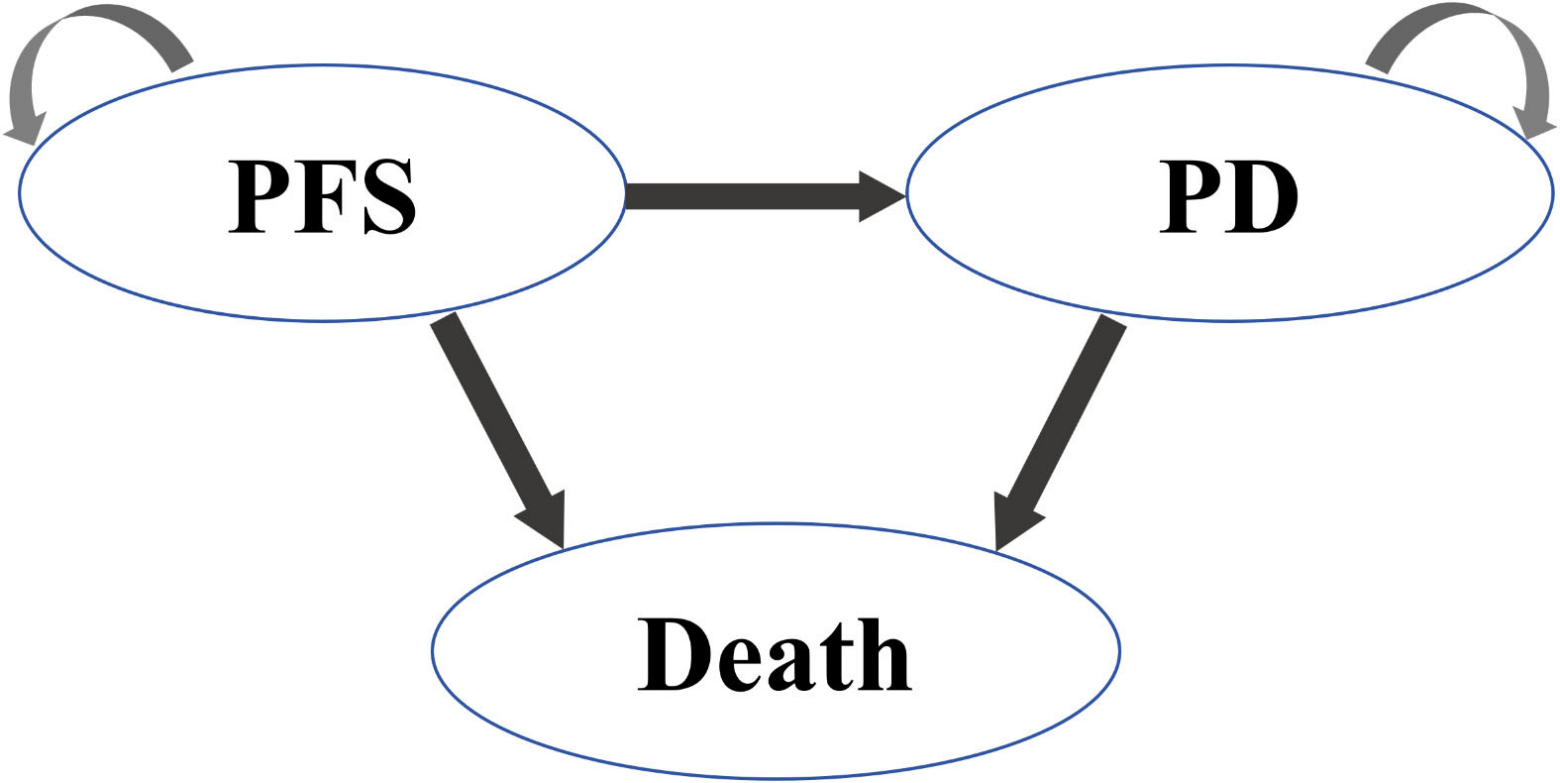
Markov model.

The model comprised three mutually exclusive health states: PFS, PD, and Death as shown in **Figure 1**. All patients initiated treatment in the PFS state, receiving durvalumab (1,500 mg IV every 4 weeks) or placebo until disease progression or unacceptable toxicity. Upon progression to PD, patients received second-line platinum-etoposide rechallenge (administered to 66.2% of progressed patients) or topotecan, followed by best supportive care (BSC).

### 2.3 Cost and utility data

The model incorporates the following direct medical costs: drug costs, including durvalumab (1500 mg per infusion at $7,631.74), simulated costs for the placebo group, and chemotherapy drugs such as topotecan ($72.17 per cycle), carboplatin ($87.94 per cycle), and etoposide ($266.67 per cycle). Treatment management-related costs cover durvalumab infusion procedures, laboratory tests ($134.36 per cycle), and imaging examinations ($140.65 per cycle) for monitoring treatment efficacy and safety. Adverse effects were only considered for those graded above 3 and with a probability of >5%, because the impact of milder adverse effects was relatively small. Post-progression treatment is divided into first-line therapy (a platinum-etoposide rechallenge regimen used by 66.2% of patients) and second-line therapy (standard regimens such as topotecan), along with best supportive care in the terminal phase ($327.46 per cycle). Palliative care costs ($2,549.63 per cycle) are used for symptom relief in the advanced stages, with end-of-life care costs included therein. In this study, in accordance with the recommendations of the Chinese Guidelines for Pharmacoeconomic Evaluation 2020, this study applied a 5% annual discount rate was applied for future health utility and cost, with a discount rate variation range of 0% to 8% (15). And all costs were converted to a unified currency using the average exchange rate of 1 USD = 7.11 CNY, based on exchange rates from 1 January 2024 to 31 October 2024 (16) (**Table 1**).

**Table 1:** Cost and model parameters.

Parameters	Base value	Lower limit	Upper limit	Distribution	Reference
Cost					
Durvalumab	7,631.74	6,105.39	9,158.09	Gama	NRDL (17).
Topotecan	72.17	57.74	86.61	Gama	NRDL (17).
Carboplatin	87.94	70.35	105.53	Gama	NRDL (17).
Etoposide	266.67	213.33	320.00	Gama	NRDL (17).
Best supportive care	327.46	261.97	392.95	Gama	DRG tariffs (18).
Laboratory test	134.36	107.49	161.23	Gama	DRG tariffs (18).
Imaging examination	140.65	112.52	168.78	Gama	DRG tariffs (18).
Palliative care	2,549.63	2,039.71	3,059.56	Gama	DRG tariffs (18).
Cost of AEs					
Rash	400.00	320.00	480.00	Gama	Yue et al. (19)
Pulmonary embolism	992.26	793.81	1,190.72	Gama	Zhang et al. (20)
Anemia	497.41	397.93	596.89	Gama	Qi et al. (21)
Fatigue	188.20	150.56	225.84	Gama	Georfieva et al. (22)
Diarrhea	5.18	4.14	6.22	Gama	Zhang et al. (23)
Hypertension	14.73	11.78	17.67	Gama	Guan et al. (24)
Utility					
PFS	0.673	0.528	0.808	Beta	Kang et al. (25–28)
PD	0.473	0.378	0.568	Beta	Kang et al. (25–28)
Disutility of AEs					
Rash	0.03	0.02	0.04	Beta	Ward et al. (29)
Pulmonary embolism	0.20	0.16	0.24	Beta	Zhang et al. (20)
Anemia	0.04	0.03	0.05	Beta	Liu et al. (30)
Fatigue	0.01	0.008	0.012	Beta	Georfieva et al. (22)
Diarrhea	0.39	0.27	0.76	Beta	Zhang et al. (23)
Hypertension	0.04	0.03	0.05	Beta	NADEES et al. (31)
Discount rate					
Discount rate	5.00%	0	8.00%	Beta	(15)

This period was chosen because it represents the most recent stable interval prior to model construction, consistent with the 2024 per capita GDP data used for willingness-to-pay thresholds, and reflects the official average central parity rates published by SAFE, thereby minimizing the impact of short-term exchange rate fluctuations.”

### 2.4 Model survival and transition probabilities

The OS and PFS data for both durvalumab and placebo groups were digitally extracted using GetData Graph Digitizer software (version 2.26). These survival curves were subsequently reconstructed and analyzed using R statistical software. To model the time-to-event data, we evaluated multiple parametric survival distributions, including expotional, gamma, gengamma, gompertz, weibull, weibull-PH, log-logistic, and log-normal distributions. Model selection was based on optimal goodness-of-fit criteria, with the final survival function chosen according to the lowest Akaike information criterion (AIC) and Bayesian information criterion (BIC) values (32, 33). Comprehensive model diagnostics are provided in **Supplementary Tables 1**, **2**. The fitted survival curves are presented in **Supplementary Figures S1**, **S2** for visual comparison between the two treatment strategies. The shape (λ) and scale (γ) parameters for the selected survival function were derived directly from the R software (version 4.3.3) output.

### 2.5 Uncertainty analysis

#### 2.5.1 Sensitivity analysis

To evaluate the robustness of the model outcomes, we conducted both one-way sensitivity analysis and probabilistic sensitivity analysis. The one-way sensitivity analysis identified the most influential parameters, and results were presented in a tornado diagram. Probabilistic sensitivity analysis was then performed by assigning predefined distributions to all parameters and running 1,000 Monte Carlo simulations. The outcomes were illustrated using scatter plots and cost-effectiveness acceptability curves. Together, these analyses revealed that drug price and utility values were the dominant drivers of cost-effectiveness uncertainty, highlighting the importance of these parameters for future policy evaluation.

#### 2.5.2 Scenario analysis

We established three distinct scenarios to simulate changes in benefits under different circumstances. Affected by factors such as medical insurance negotiations and policy support, the prices of innovative drugs included in the medical insurance catalog typically decrease by 10% to 50% through renewal negotiations and dynamic adjustments. This study is based on the average of this reduction range, meaning a further 30% reduction from the original price of durvalumab. Since utility values may vary across different studies and have a significant impact on cost-utility analysis results, this study refers to other relevant literature to modify the utility values for PFS and PD states, thereby further validating the robustness of the model. The adjusted parameters and distributions after changing the utility values are presented in the **Table 2**. Additionally, to align with the ADRIATIC trial protocol and ensure the accuracy of our cost calculations, we have capped the treatment duration of durvalumab at 24 months. This means that in our cost calculations, we only account for the costs of durvalumab treatment for up to 24 months, as per the trial’s specified maximum duration. This approach prevents the overestimation of costs that could arise from assuming treatment beyond the clinically validated timeframe.

**Table 2:** Parameters and distribution of changed utility value.

Utility value	Basis value	Range		Source
		Min	Max	
PFS value	0.86	0.65	0.97	Shen Y et al. (34)
PD value	0.77	0.54	0.80	Shen Y et al. (34)

Given the suboptimal cost-effectiveness of durvalumab observed in our analysis, we also conducted a threshold price analysis to explore the price at which durvalumab would become cost-effective under different willingness-to-pay (WTP) thresholds. Specifically, we evaluated the cost-effectiveness of durvalumab at both one times (1x) and three times (3x) the WTP threshold. Our analysis aimed to determine the price points at which durvalumab would fall within the commonly accepted cost-effectiveness ratios, thereby providing valuable insights for policymakers and healthcare decision-makers.

#### 2.5.3 Subgroup analysis

The results of the subgroup analysis are presented in **Table 3**. At a WTP threshold of $12,569.82 per QALY, the subgroup with the highest probability of being cost-effective was the carboplatin-etoposide subgroup in the Previous chemotherapy category, with a cost of $186,589.57 and an ICER of $167,476.98 per QALY. This was followed by the Europe subgroup in the Geographic region category, with a cost of $175,107.56 and an ICER of $181,930.53 per QALY. A similar trend was observed at a WTP threshold of three times the per capita GDP of China.

**Table 3:** CEA results of base-case.

Treatment	Total costs	Total QALYs	Incre costs	Incre QALYs	ICER	NMB	INMB
Placebo group	63,274.61	1.80				-46,249.06	
Durvalumab group	171,884.05	2.24	108,609.45	0.44	245,591.59	-153,643.40	-107,394.34

## 3 Results

### 3.1 Base case results

The **Table 4** presents the basic case evaluation results of the cost-effectiveness of the two treatment regimens. The total cost for the placebo group is $63,274.61, with a total of 1.80 QALYs; whereas for durvalumab, the total cost amounts to $171,884.05, and the total QALYs reach 2.24. By calculating the ICER, it is determined that an additional expenditure of approximately $245,591.59 is required to gain one extra QALY. Additionally, the NMB was calculated, resulting in -$46,249.06 for the placebo group and -$153,643.40 for the durvalumab group. This indicates that the placebo group yields a higher net benefit compared to durvalumab, as a larger NMB is more favorable. The INMB for durvalumab versus placebo is -$107,394.34, which further demonstrates that the durvalumab group provides a negative incremental net benefit and is not economically advantageous relative to the placebo group. Based on these findings-including the high ICER value, the superior NMB of the placebo group, and the negative INMB-the placebo group is the more cost-effective option at the given WTP threshold implicit in the analysis.

**Table 4:** Results of the analysis under different scenarios.

Treatment	Total costs (qalys)	Incre costs (qalys)	ICER	NMB	INMB
Scenario analysis 1: cost value sensitivity results						
Placebo group	63,274.61			-46,512.36	
Durvalumab group	139,025.89	75,751.28	171,291.54	-118,974.78	-72,462.41
Scenario analysis 2: utility value sensitivity results						
Placebo group	(2.47)			-37,184.27	
Durvalumab group	(3.04)	(0.57)	191,017.07	-142,516.19	-105,331.92
Scenario analysis 3: 24 months sensitivity results						
Placebo group	30,143.57			-20,462.27	
Durvalumab group	136,768.70	106,625.13	1,337,187.91	-130,712.35	-110,250.07
Scenario analysis 4: results of value-based pricing analysis						
Durvalumab ($451.28/cycle) group	68,833.41	5,558.81	12,295.73	-46,211.25	156.03
Durvalumab ($1,225.95/cycle) group	79,951.12	16,676.51	36,886.98	-183,759.06	-37,715.96

### 3.2 Results of sensitivity analysis

**Figure 2:**
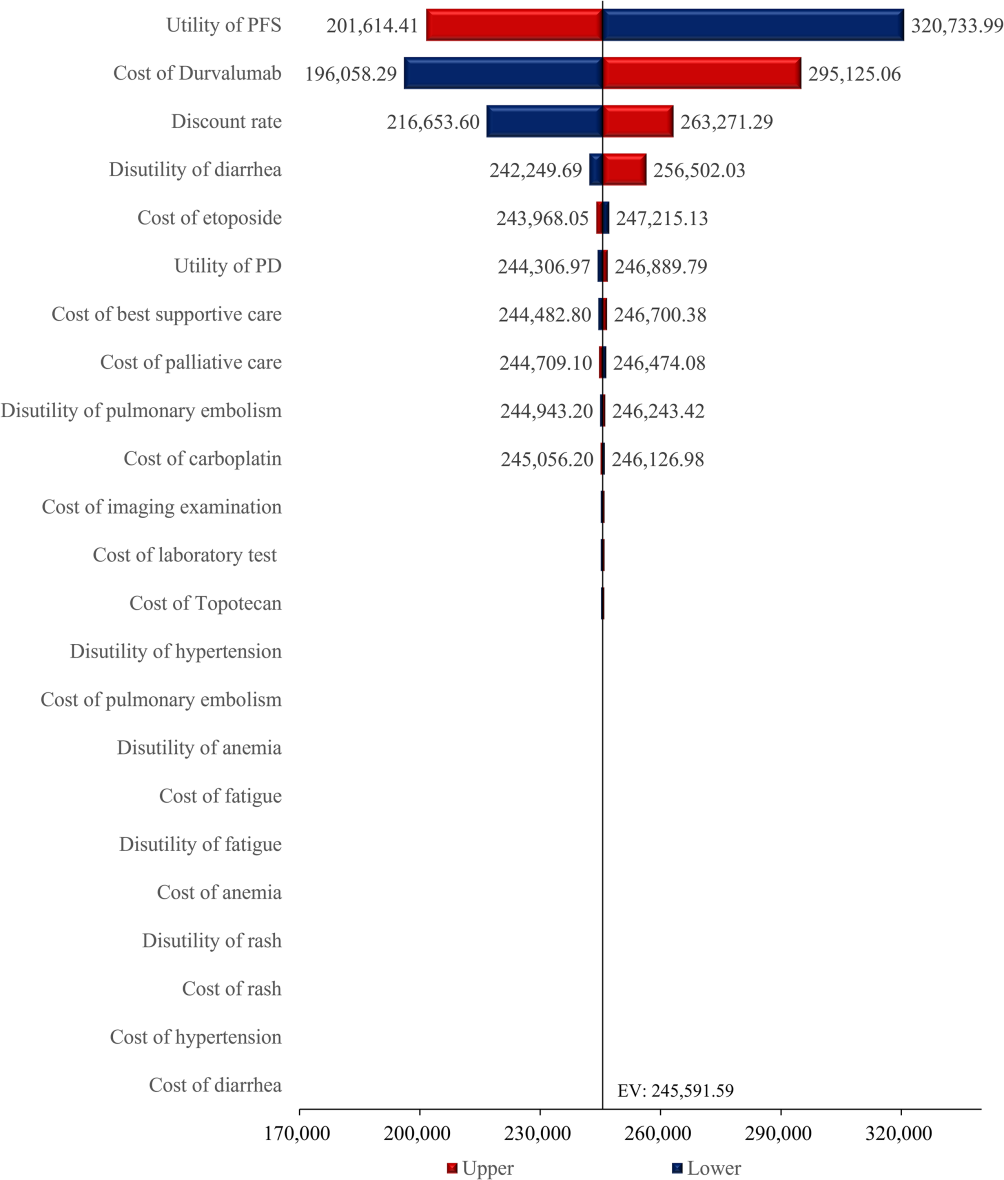
Tornado diagram.

This deterministic sensitivity analysis, as presented in the results table, demonstrated that among all evaluated parameters, variations in the utility for PFS exerted the most profound influence on model outcomes, with a fluctuation magnitude of $119,119.58 per QALY units between its lower bound ($201,614.41 per QALY) and upper bound ($320,733.99 per QALY), establishing it as the primary sensitivity driver (**Figure 2**). The cost of durvalumab constituted the second most critical factor, exhibiting a substantial variation of $99,066.78 per QALY units across its tested range ($196,058.29 per QALY - $295,125.06 per QALY), underscoring its pivotal role in cost-effectiveness conclusions. The discount rate ranked third in sensitivity impact, generating a variation of 46,617.70 units ($216,653.60 per QALY - $263,271.29 per QALY), indicating significant temporal effects on economic evaluations. Other parameters-including drug costs, healthcare service costs, progressive disease utility-collectively and etc. induced smaller-scale fluctuations in model outputs.

### 3.3 Probabilistic sensitivity analysis

**Figure 3:**
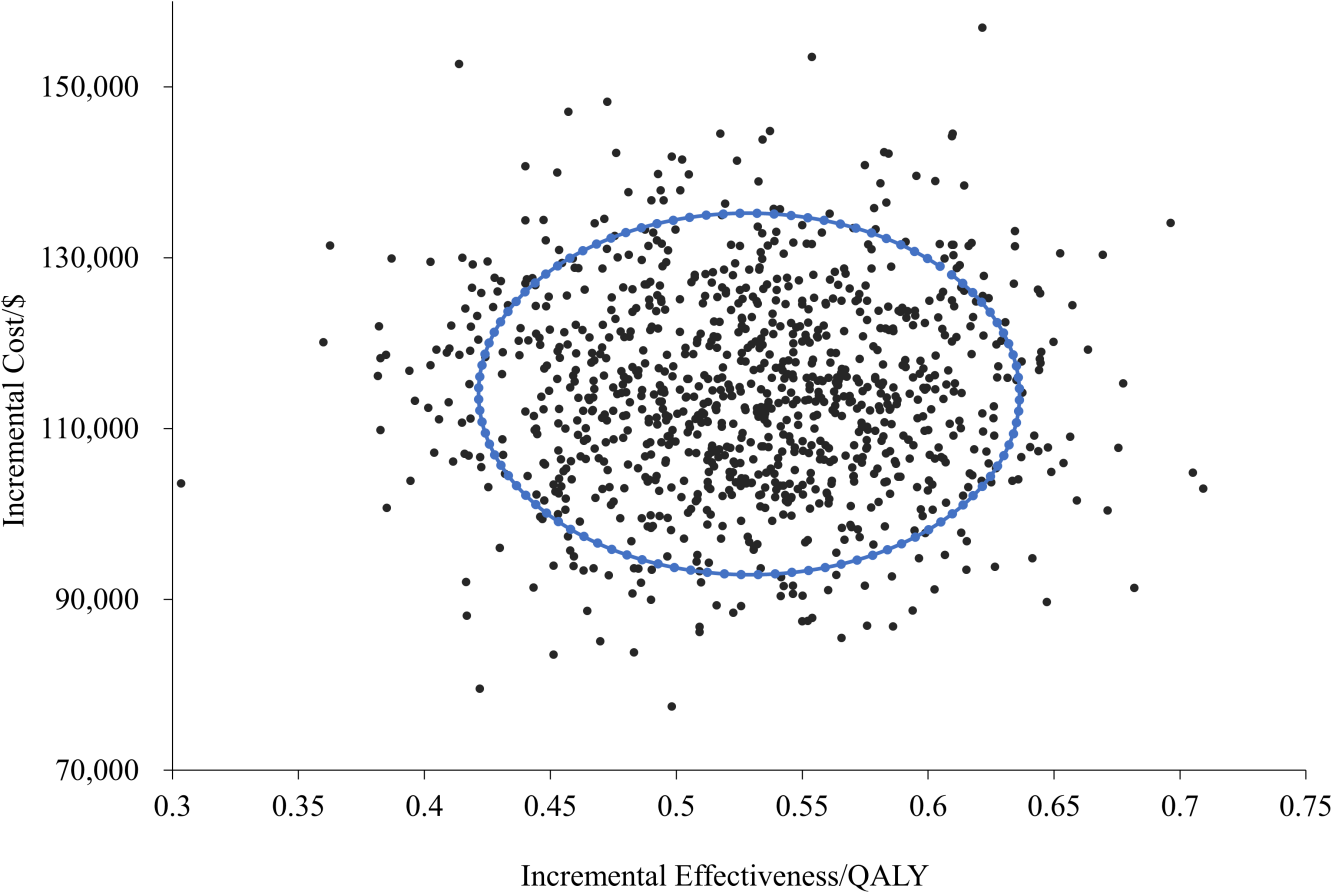
Probabilistic sensitivity analysis results of the different treatments.

In **Supplementary Files S3**, **S4**, we have provided the detailed PSA parameter settings and partial results of the Monte Carlo simulations. As shown by the cost-effectiveness scatter plot (**Figure 3**), all the scatter points are located in the fourth quadrant, indicating that in the treatment of LS-SCLC, the durvalumab treatment regimen, compared with the placebo regimen, can achieve better health outcomes at a higher cost in all simulated scenarios.

**Figure 4:**
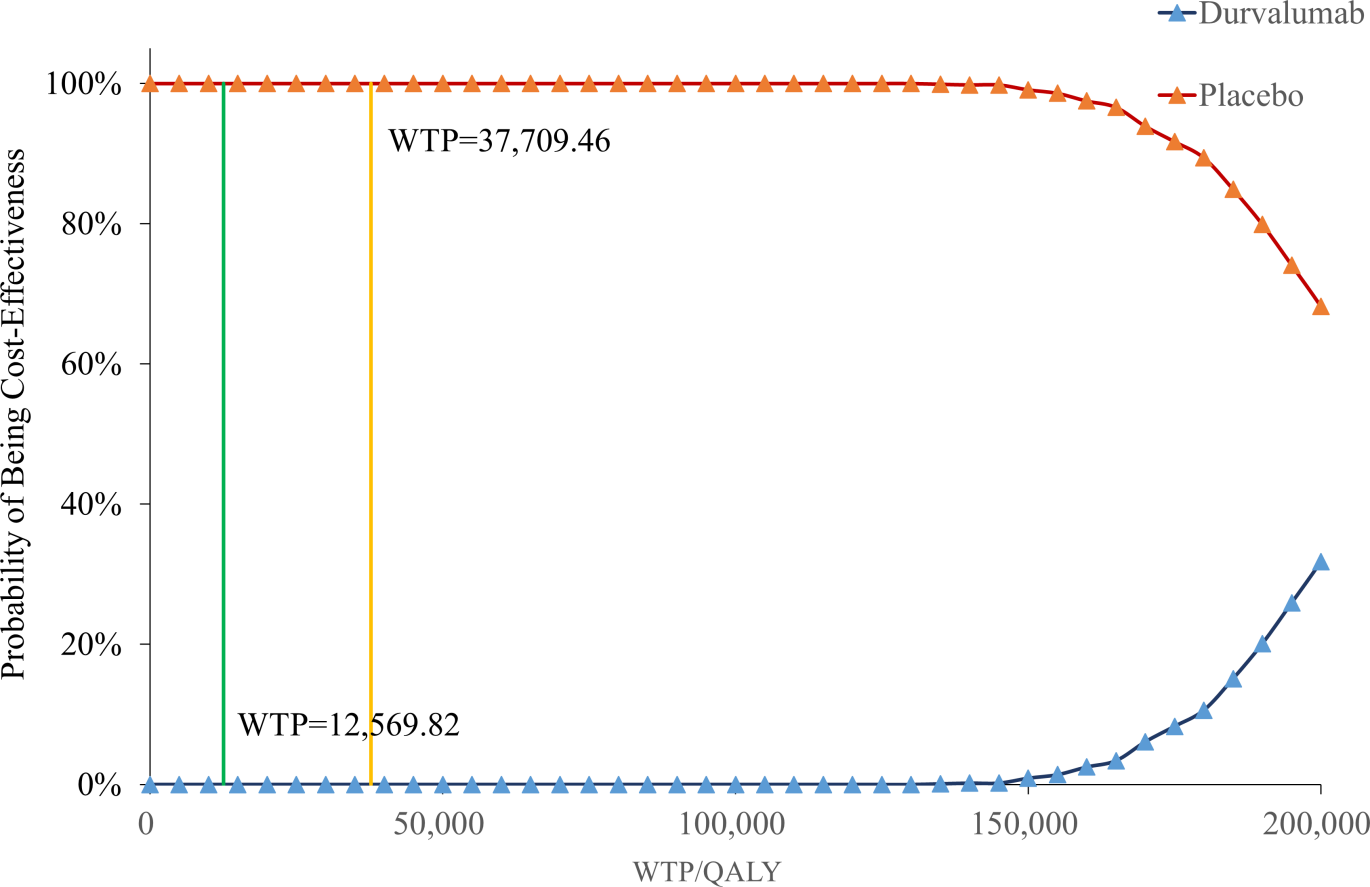
Cost-effectiveness acceptability curve.

**Figure 4** showed that when the willingness-to-pay (WTP) threshold was below $37,709.46, the cost-effectiveness probability of placebo was constantly 100%, indicating that it had a significant economic advantage in this range. When the WTP threshold reached or exceeded $130,000.00, the probability of placebo gradually decreased, while the probability of durvalumab increased.

### 3.4 Expected value of perfect information analysis

**Figure 5:**
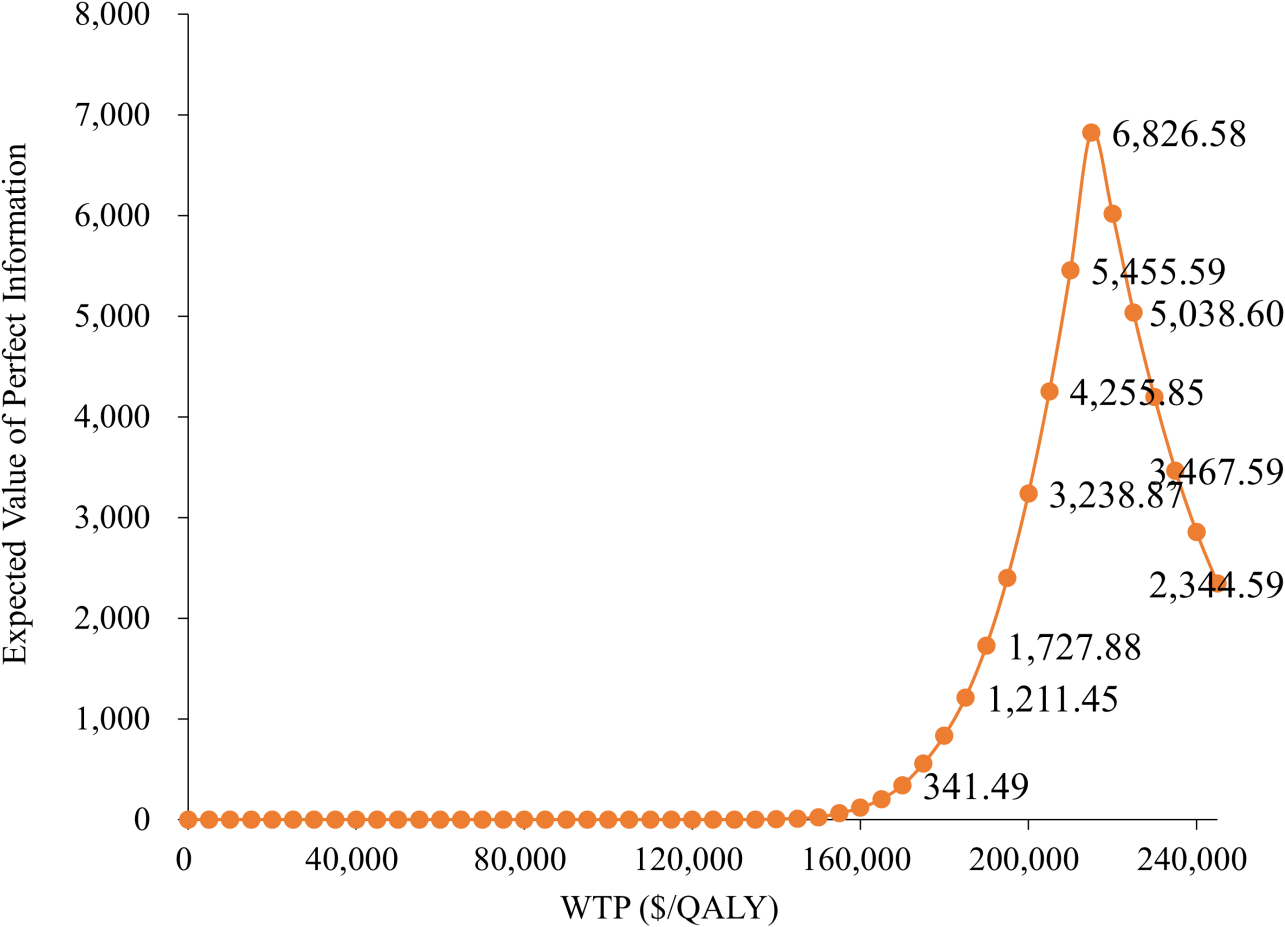
Expected value of perfect information.

**Figure 5** demonstrates that the Expected Value of Perfect Information (EVPI) is relatively low at lower WTP thresholds but increases significantly as the WTP approaches $200,000 per QALY, peaking at $6,826.58. This indicates that under high WTP scenarios, the uncertainty in model parameters becomes more critical, highlighting the increased decision risk associated with higher payment thresholds.

### 3.5 Results of scenario analysis

The results of scenario analysis 1 indicate that under conditions of changing cost values, the cost-effectiveness of the treatment with durvalumab does not change. The durvalumab group also shows improvement in health outcomes compared to the placebo group at a different utility value (lower than 3 times WTP) (**Table 4**). Scenario analysis 3 indicates that this adjustment does not significantly alter the cost-effectiveness profile of durvalumab, supporting the robustness of our base-case analysis.

Scenario analysis 4: The value-based pricing analysis presented in **Table 4** shows that when the ICER of durvalumab is $12,295.73 per QALY, the price of durvalumab per cycle needs to be reduced to $451.28. When the ICER is adjusted to three times the threshold, the price of durvalumab per cycle needs to be reduced to $1,225.95 (**Table 4**).

### 3.6 Subgroup analysis

The results of the subgroup analysis are presented in **Table 4**. At a WTP threshold of $12,569.82 per QALY, the subgroup with the highest probability of being cost-effective was the carboplatin-etoposide subgroup in the Previous chemotherapy category, with a cost of $187,637.23 and an ICER of $175,714.96 per QALY. This was followed by the Europe subgroup in the Geographic region category, with a cost of $176,166.22 and an ICER of $192,813.78 per QALY. A similar trend was observed at a WTP threshold of three times the per capita GDP of China.

## 4 Discussion

This study, based on the data from the ADRIATIC Phase III clinical trial, constructed a Markov model adapted to the Chinese health economic context to systematically evaluate the cost-effectiveness of durvalumab as consolidation therapy for LS-SCL following concurrent chemoradiotherapy. The results indicate that although durvalumab significantly prolonged patients’ PFS and OS, and increased QALYs, its ICER is much higher than the widely accepted willingness-to-pay threshold, suggesting poor cost-effectiveness under the current pricing strategy and potentially limiting its widespread clinical application in China.

Further sensitivity analysis revealed that drug costs, health utility values, and discount rates are the key parameters affecting the model outcomes. Among them, fluctuations in the price of durvalumab were most sensitive to changes in ICER, demonstrating that drug pricing remains the core factor constraining its cost-effectiveness. Additionally, variations in the upper and lower limits of PFS utility values also had a significant impact on the results, indicating that improvements in quality of life are equally important for enhancing cost-effectiveness.

Notably, the probabilistic sensitivity analysis results showed that the cost-effectiveness advantage of durvalumab is significantly dependent on a higher WTP threshold. When the willingness-to-pay is below $150,000 per QALY, the probability of its cost-effectiveness is close to zero; even with a high payment threshold, although there is some improvement, it remains significantly lower than that of the placebo group. This trend suggests that durvalumab is more suitable for healthcare systems with stronger payment capabilities or for specific high-value patient groups who are extremely sensitive to survival benefits. In contrast, the placebo maintains a strong cost-effectiveness advantage under conventional payment standards.

Moreover, the EVPI analysis indicated that model uncertainty sharply increases in the high willingness-to-pay range, implying that if payment standards are further raised in the future, there will be a greater risk of resource allocation. Therefore, it is necessary to conduct more in-depth research on the sensitivity parameters under high willingness-to-pay scenarios (such as long-term survival data and drug cost structure) to reduce potential losses caused by uncertainty and provide more robust evidence for health insurance policy formulation.

Our study has the following limitation. Although the comparator we selected is consistent with the ADRIATIC trial, differences in real-world practice, such as the use of PCI and follow-up imaging, may affect the generalizability of our study results. To better assess the impact of these differences on cost-effectiveness, future studies could consider incorporating these real-world practices into the analysis to more comprehensively reflect the actual effects in clinical practice.

In summary, although the overall cost-effectiveness is currently insufficient, it may still be possible to improve the cost-effectiveness and accessibility of durvalumab under certain conditions through subgroup strategies targeting high-risk patients or the introduction of price optimization mechanisms (such as risk-sharing agreements).

## 5 Conclusion

This study demonstrates that durvalumab, as consolidation therapy for limited-stage small cell lung cancer following concurrent chemoradiotherapy, has clear survival benefits and improvements in health-related quality of life. However, its cost-effectiveness remains inadequate under the current pricing system and payment capacity. The core influencing factors include drug pricing, health utility, and uncertainty in long-term efficacy data.

Future policies could consider optimizing the cost structure through differentiated health insurance payment strategies, price negotiation mechanisms, and risk-sharing models, while also strengthening the collection and assessment of real-world efficacy and cost data to further clarify its applicable scenarios within the Chinese healthcare system. Only by achieving a balance between clinical value and economic burden can durvalumab truly realize its widespread application in the treatment of limited-stage small cell lung cancer.

## Glossary

SCLC: Small cell lung cancer
LS-SCLC: Limited-stage small cell lung cancer
ES-SCLC: Extensive-stage small cell lung cancer
OS: Overall survival
PFS: Progression-free survival
mNSCLC: Non-small cell lung cancer
EFS: Event-free survival
pCR: Pathological complete response rate
NMPA: The National Medical Products Administration
NHSA: The National Healthcare Security Administration
PD: Progressive disease
ICER: Incremental cost-effectiveness ratio
QALYs: Quality-adjusted life-years
EVPI: Expected value of perfect information
Lys: Life years
BSC: Best supportive care
