# Supplementary material for: Durvalumab consolidation therapy in patients with stage III small cell lung cancer after concurrent chemoradiation: a China-based cost-effectiveness analysis
Source: Front Oncol. 2025 Nov 5;15:1643022. doi: 10.3389/fonc.2025.1643022 (PMC12626786; doi:10.3389/fonc.2025.1643022)
Supplement: Supplementary file 1 [file DataSheet1.pdf]

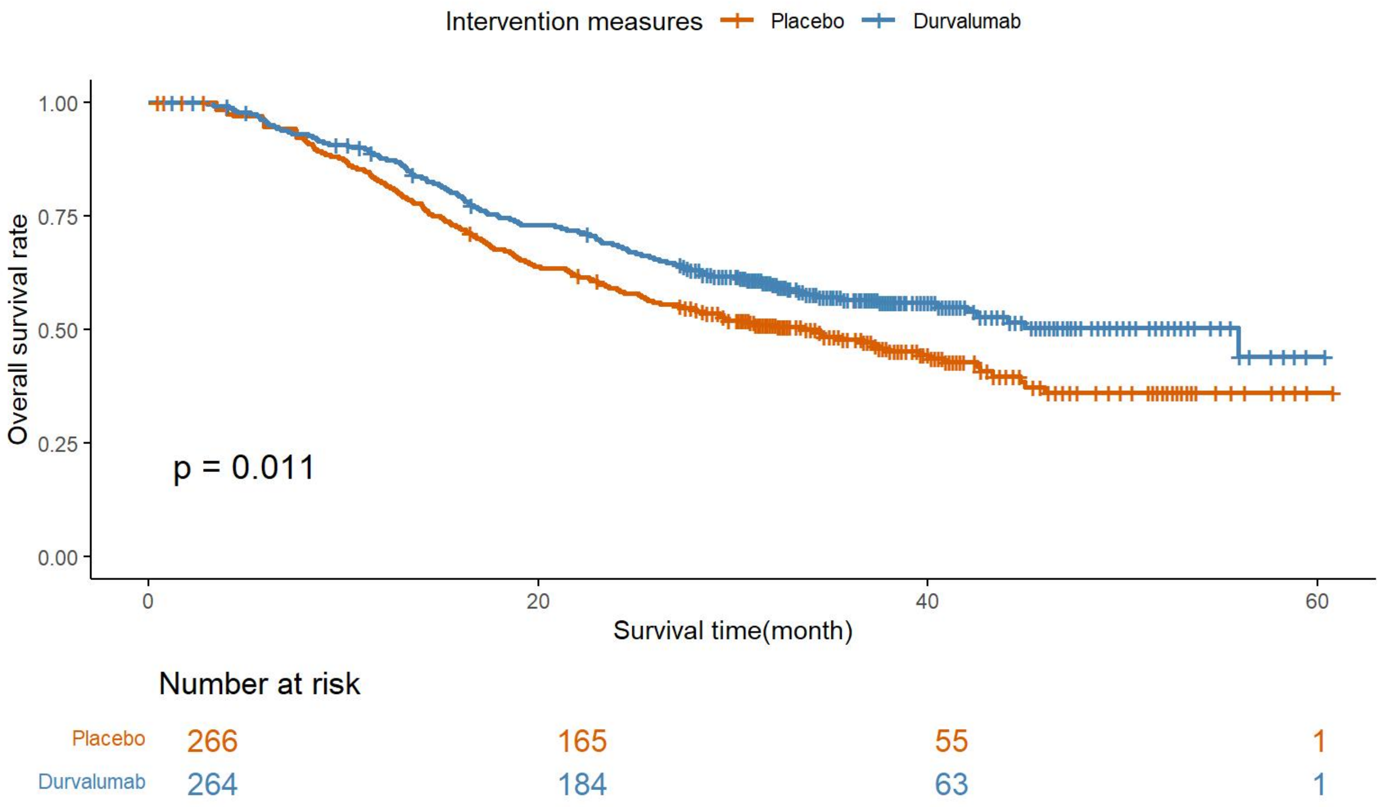

Figure S1 Original OS curve.

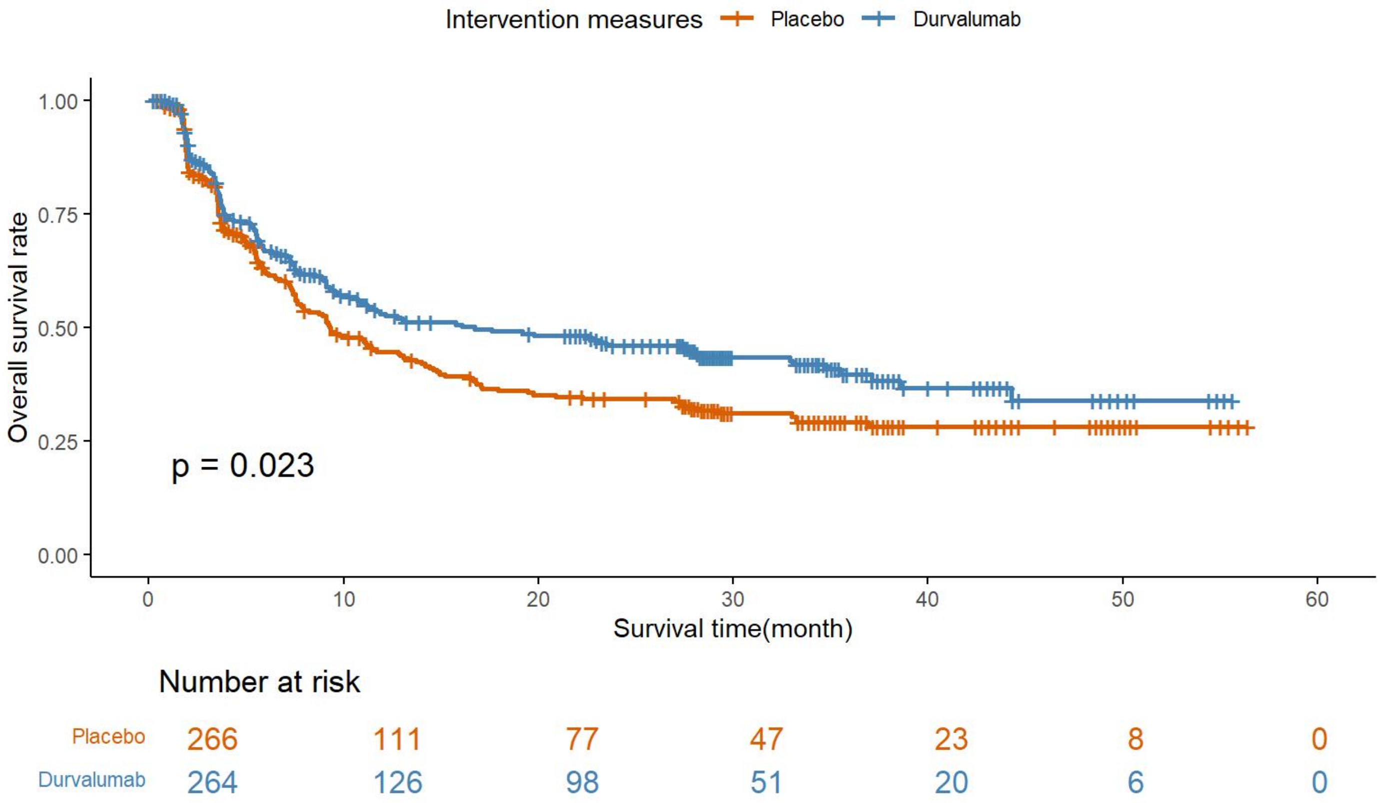

Figure S2 Original PFS curve.

Table S1 Goodness-of-fit under different distributions of survival curves between the three patient groups.

Distribution function	Fit metrics	Expotional	Gamma	Gengamma	Gompertz	Weibull	WeibullPH	Log-logistic	Log-normal
OS curve of the durvalumab	AIC	1191.204	1184.267	1168.843	1192.981	1186.803	1186.803	1179.265	1172.321
	BIC	1194.780	1191.419	1179.571	1200.133	1193.955	1193.955	1186.417	1179.472
OS curve of the placebo	AIC	1424.974	1412.653	1391.536	1426.330	1416.939	1416.939	1404.280	1396.310
	BIC	1428.558	1419.820	1402.286	1433.497	1424.106	1424.106	1411.447	1403.477
PFS curve of the durvalumab	AIC	1231.739	1219.951	1130.493	1181.299	1213.205	1213.205	1192.792	1179.423
	BIC	1235.315	1227.103	1141.221	1188.451	1220.357	1220.357	1199.944	1186.575
PFS curve of the placebo	AIC	1402.456	1392.106	1301.242	1333.611	1382.598	1382.598	1346.720	1336.513
	BIC	1406.040	1399.273	1311.993	1340.778	1389.765	1389.765	1353.887	1343.680

Table S2 Parameters of parametric models for virtual time-to-event data.

Treatment regimens	Endpoint	Distribution	Distribution information
Durvalumab group	OS	Gamma	mu: 3.30523<br>sigma: 1.31470<br>Q: -1.14386
	PFS	Gamma	mu: 3.073831<br>sigma: 1.125275<br>Q: -0.982798
Placebo group	OS	Generalised gamma	mu: 1.07758<br>sigma: 1.01657<br>Q: -3.29786
	PFS	Generalised gamma	mu: 1.47978<br>sigma: 1.26077<br>Q: -1.68207

Table S3 Probabilistic Sensitivity Analysis Parameters

Parameter	Mean	SE	Distribution	α	β

Cost of durvalumab	7,631.74	778.75	Gama	96.04	79.46
Cost of topotecan	72.17	7.36	Gama	96.03	0.75
Cost of carboplatin	87.94	8.97	Gama	96.04	0.92
Cost of etoposide	266.67	27.21	Gama	96.04	2.78
Cost of best supportive care	327.46	33.41	Gama	96.04	3.41
Cost of laboratory test	134.36	13.71	Gama	96.04	1.40
Cost of imaging examination	140.65	14.35	Gama	96.04	1.46
Cost of palliative care	2,549.63	260.17	Gama	96.04	26.55
Utility of PFS	0.67	0.07	Beta	28.36	13.78
Utility of PD	0.47	0.05	Beta	49.71	55.39
Discount rate	0.05	-	Beta	-	-

Table S4 Results of the first 50 output of 1,000 Monte Carlo simulations

Tmes	Costof placebo	QALY of placebo	Cost of durvalumab	QALY of durvalumab	Incre QALYs	Incre costs
1	78,919.56	1.78	184,907.16	2.22	0.45	105,987.59
2	63,471.53	2.13	164,886.11	2.71	0.58	101,414.58
3	73,393.26	1.61	184,495.90	2.02	0.41	111,102.65
4	72,149.61	1.93	176,501.85	2.41	0.49	104,352.24
5	75,034.52	1.93	201,806.50	2.42	0.49	126,771.98
6	69,200.47	1.90	164,275.20	2.39	0.50	95,074.73
7	65,737.58	2.12	198,622.36	2.66	0.53	132,884.78
8	78,986.53	2.09	195,074.80	2.66	0.57	116,088.27
9	74,457.29	2.15	189,642.96	2.73	0.58	115,185.68
10	63,502.94	2.25	186,536.09	2.85	0.60	123,033.16
11	76,489.81	1.64	181,199.13	2.07	0.43	104,709.32
12	73,008.11	1.70	184,493.75	2.14	0.44	111,485.64
13	71,228.78	1.78	198,027.41	2.22	0.44	126,798.63
14	72,718.64	2.17	186,906.44	2.78	0.61	114,187.81

15	77,863.73	2.14	187,692.32	2.74	0.60	109,828.58
16	67,240.14	1.85	151,073.09	2.34	0.48	83,832.96
17	70,647.05	1.94	183,781.77	2.45	0.51	113,134.73
18	71,845.33	2.25	175,300.31	2.85	0.60	103,454.98
19	75,876.33	1.98	174,480.20	2.47	0.49	98,603.87
20	73,125.58	1.97	166,434.97	2.48	0.51	93,309.39
21	73,576.32	2.02	185,773.70	2.54	0.52	112,197.38
22	69,982.27	1.97	187,843.98	2.49	0.53	117,861.71
23	73,770.58	2.07	196,519.89	2.64	0.58	122,749.31
24	78,527.65	1.88	195,283.62	2.36	0.48	116,755.97
25	78,433.45	2.19	197,656.56	2.77	0.58	119,223.11
26	68,623.78	1.96	180,247.53	2.51	0.56	111,623.75
27	73,152.14	1.98	178,619.59	2.55	0.57	105,467.44
28	78,150.55	2.06	174,594.91	2.64	0.58	96,444.36
29	75,061.67	2.02	177,174.15	2.54	0.52	102,112.49
30	75,335.93	1.94	183,530.97	2.43	0.49	108,195.04
31	71,178.91	2.04	189,180.12	2.58	0.54	118,001.21
32	68,382.22	2.14	184,492.06	2.71	0.57	116,109.84
33	68,268.26	1.93	177,607.02	2.47	0.54	109,338.77
34	74,175.89	1.99	177,526.36	2.53	0.54	103,350.47
35	75,038.93	1.79	209,106.12	2.26	0.47	134,067.19
36	73,749.02	2.08	185,261.17	2.65	0.57	111,512.15
37	72,627.58	2.03	180,560.58	2.56	0.53	107,933.00
38	72,631.94	2.20	192,833.99	2.81	0.61	120,202.04
39	76,511.07	2.20	188,686.00	2.78	0.58	112,174.93
40	79,090.14	1.95	194,183.33	2.44	0.49	115,093.19
41	74,958.92	1.93	191,717.14	2.41	0.48	116,758.22
42	69,468.30	1.82	157,603.89	2.24	0.42	88,135.59
43	73,165.44	2.12	181,895.09	2.71	0.59	108,729.65
44	69,670.69	1.98	177,284.75	2.50	0.52	107,614.07
45	72,165.61	2.14	186,973.02	2.68	0.54	114,807.42
46	68,480.62	1.91	196,796.44	2.42	0.51	128,315.82

47	73,850.94	2.01	182,396.34	2.51	0.50	108,545.40
48	77,776.21	1.72	186,549.12	2.15	0.43	108,772.90
49	71,266.44	2.20	172,507.68	2.76	0.56	101,241.24
50	73,526.51	2.04	218,374.92	2.58	0.54	144,848.41
